# Editorial: Behçet's Disease: Epidemiology, Etiopathogenesis, Diagnosis and Treatment

**DOI:** 10.3389/fmed.2021.794874

**Published:** 2021-11-05

**Authors:** Erkan Alpsoy, Pietro Leccese, Tulin Ergun

**Affiliations:** ^1^Department of Dermatology and Venereology, School of Medicine, Akdeniz University, Antalya, Turkey; ^2^Rheumatology Institute of Lucania (IRel) and the Rheumatology Department of Lucania, San Carlo Hospital of Potenza and Madonna delle Grazie Hospital of Matera, Potenza and Matera, Italy; ^3^Department of Dermatology, Marmara University School of Medicine, Istanbul, Turkey

**Keywords:** etiopathogenesis, diagnosis, treatment, Behçet's disease, clinic, epidemiology

Behçet's disease (BD) is a chronic, relapsing inflammatory disease of unknown etiology with the clinical features of mucocutaneous lesions and ocular, vascular, articular, neurologic, gastrointestinal, urogenital, pulmonary, and cardiac involvement. The disease has the highest incidence in “Silk Road” populations but has global distribution. Although the number of effective drugs used in the disease's treatment has increased in recent years, BD is still associated with severe morbidity because of mainly mucocutaneous, articular and ocular symptoms and increased mortality because of large vessel, neurological, gastrointestinal and cardiac involvement. Although the gender distribution is roughly equal, BD runs a more severe course in the male gender. Again, the disease course is more severe in the young.

In this special Research Topic, we try to deal with BD in all aspects due to its multisystemic nature. Therefore, we think that this topic will attract the attention of many disciplines. We aim to bring the most recent developments in BD to a close. We particularly focus on the current views and state of knowledge regarding etiopathogenesis, clinical characteristics and spectrum, and therapeutic approaches.

Diagnosis in most patients can be made clinically only on the basis of mucocutaneous symptoms, which figure prominently in the presentation and diagnosis and are considered the hallmarks of the disease. Nakamura et al., in the article “Mucocutaneous manifestations of Behcet's disease,” emphasize the importance of mucocutaneous manifestations, which precede severe organ involvement in most patients. Therefore, familiarity with these symptoms is imperative for early diagnosis and prevention of potentially serious organ involvement through appropriate management.

Although the main and the most frequent manifestations of the disease are recurrent oral and genital ulcers, cutaneous lesions, ocular inflammation, and arthritis, major vessel and life- or organ-threatening involvement of internal organs can be seen during the course of BD. Kötter and Lötscher provide an overview of these less frequently documented vascular, neurologic, gastrointestinal and musculoskeletal manifestations of the disease, which can nevertheless be decisive in terms of prognosis and differential diagnosis.

Although the disease usually starts around the third or fourth decade of life, 15–20% of all BD patients develop in childhood. Yildirim et al. overview the definition, clinical findings, epidemiology, etiopathogenesis, and treatment of pediatric BD, which differs from adult BD not only by the age of onset but also by the frequency and distribution of clinical findings, disease severity, and outcome. While gastrointestinal system involvement, neurological findings, arthralgia and positive family history are more common in children, genital ulcers and vascular lesions are noted less frequently. In general, children have a better prognosis with a lower severity score and activity index.

In the “Pathergy phenomenon” title, Ergun emphasizes the importance of the skin pathergy test, a hyperreactivity response to needle induced trauma. Being one of the diagnostic criteria for BD, its positivity is affected by a wide array of factors, such as the physical properties of the needles being used, number of pricks, male gender and disease activity. In addition, controversy exists as to the sensitivity of this phenomenon which varies between geographic areas. However, the skin pathergy test may also serve as a model for investigating the inflammatory pathways involved in the etiopathogenesis of this complex disease.

The etiology of BD remains unclear. Besides genetic HLA and non-HLA predisposing associations and epigenetic influence, environmental factors are thought to contribute to the pathogenesis of the disease, and among these, infectious agents and specific microbiome alterations are considered of particular relevance in BD pathogenesis. Mattioli et al. discuss the current evidence on the main genetic, environmental and immunological factors contributing to BD development. The disease can be defined as a multifactorial disease and shares some common features with autoimmune, autoinflammatory or spondyloarthropathies (MHC-I-opathies). Advances in understanding the etiopathogenesis of BD help us to identify modifiable risk factors of BD occurrence and development of targeted treatments. Ortiz-Fernández and Sawalha provide an updated view of the genetic landscape and architecture of BD. Using large-scale genetic data available, they estimate the heritability of BD for the first time to be at least 16%. They further calculate a cumulative genetic risk score across various populations and obtain significant differences among populations' genetic risk scores. The highest genetic risk score is observed in East Asian, followed by European, and South Asian populations, while Admixed Americans and African populations show the lowest values. In the pathogenesis of the disease, environmental factors, especially infectious agents or microbiota, has long been suspected. They can trigger innate immune system-mediated inflammation sustained by adaptive (acquired) immune responses. Mumcu and Fortune describe changes in the oral environment consequently act as an infective and immune trigger. They discuss complex interactions between the oral ulcers, the oral microbiome and immune responses together with the course of oral and systemic disease manifestations in the context of the aetiologic role of oral health.

The treatment of BD is selected according to the organ involved and the severity of the involvement. Therefore, treatment should be personalized and tailored to the affected organ(s) with a multidisciplinary approach. Based on the mainly controlled studies and personal experience in clinical practice and basic research in this field, Alpsoy et al. propose a stepwise, symptom-based, algorithmic approach for the management of BD.

In conclusion, we try to explain all aspects of BD to the readers with a holistic approach with the help of very well-known names on this topic. In this Research Topic, Up-To-Date and concise writing has been preferred in each title as much as possible. In addition, evidence-based algorithmic approaches have been proposed in appropriate titles. We hope that this special issue will be helpful in the daily clinical practice of physicians dealing with BD.

## Author Contributions

All authors listed have made a substantial, direct and intellectual contribution to the work, and approved it for publication.

## Conflict of Interest

The authors declare that the research was conducted in the absence of any commercial or financial relationships that could be construed as a potential conflict of interest.

## Publisher's Note

All claims expressed in this article are solely those of the authors and do not necessarily represent those of their affiliated organizations, or those of the publisher, the editors and the reviewers. Any product that may be evaluated in this article, or claim that may be made by its manufacturer, is not guaranteed or endorsed by the publisher.

